# Evaluation of 11 years of newborn screening for maple syrup urine disease in the Netherlands and a systematic review of the literature: Strategies for optimization

**DOI:** 10.1002/jmd2.12124

**Published:** 2020-05-13

**Authors:** Kevin Stroek, Anita Boelen, Marelle J. Bouva, Monique De Sain‐van der Velden, Peter C. J. I. Schielen, Rose Maase, Henk Engel, Bernadette Jakobs, Leo A. J. Kluijtmans, Margot F. Mulder, M. E. Rubio‐Gozalbo, Francjan J. van Spronsen, Gepke Visser, Maaike C. de Vries, Monique Williams, Annemieke C. Heijboer, Evelien A. Kemper, Annet M. Bosch

**Affiliations:** ^1^ Endocrinology Laboratory, Department of Clinical Chemistry Amsterdam Gastroenterology & Metabolism, Amsterdam UMC, University of Amsterdam Amsterdam The Netherlands; ^2^ Reference Laboratory Neonatal Screening, Center for Health protection National Institute for Public Health and the Environment Bilthoven The Netherlands; ^3^ Section Metabolic Diagnostics, Department of Genetics University Medical Center Utrecht Utrecht The Netherlands; ^4^ Department of Clinical Chemistry Isala Hospital Zwolle The Netherlands; ^5^ Department of Clinical Chemistry Elisabeth‐Tweesteden Hospital Tilburg The Netherlands; ^6^ Translational Metabolic Laboratory, Department of Laboratory Medicine Radboud University Medical Center Nijmegen The Netherlands; ^7^ Department of Pediatrics, Division of Metabolic Disorders Amsterdam UMC, Vrije Universiteit Amsterdam Amsterdam The Netherlands; ^8^ Department of Pediatrics and Clinical Genetics Maastricht University Medical Center Maastricht The Netherlands; ^9^ Division of Metabolic Disorders, Beatrix Children's Hospital University Medical Center Groningen, University of Groningen Groningen The Netherlands; ^10^ Wilhelmina Children's Hospital University Medical Center Utrecht Utrecht The Netherlands; ^11^ Department of Pediatrics, Division of Metabolic Disorders Radboud University Medical Center Nijmegen The Netherlands; ^12^ Center for Lysosomal and Metabolic diseases, Department of Pediatrics Erasmus Medical Center Rotterdam The Netherlands; ^13^ Endocrinology Laboratory, Department of Clinical Chemistry Amsterdam Gastroenterology & Metabolism, Amsterdam UMC, Vrije Universiteit Amsterdam Amsterdam The Netherlands; ^14^ Department of Clinical Chemistry IJsselland Hospital Capelle aan den IJssel The Netherlands; ^15^ Department of Pediatrics, Division of Metabolic Disorders Amsterdam UMC, University of Amsterdam Amsterdam The Netherlands

**Keywords:** dried blood spots, leucine, maple syrup urine disease, mass‐spectrometry, newborn screening, positive predictive value, valine

## Abstract

Maple syrup urine disease (MSUD) leads to severe neurological deterioration unless diagnosed early and treated immediately. We have evaluated the effectiveness of 11 years of MSUD newborn screening (NBS) in the Netherlands (screening >72 hours, referral if both total leucine (Xle) and valine ≥400 μmol/L blood) and have explored possibilities for improvement by combining our data with a systematic literature review and data from Collaborative Laboratory Integrated Reports (CLIR). Dutch MSUD NBS characteristics and accuracy were determined. The hypothetical referral numbers in the Dutch population of additional screening markers suggested by CLIR were calculated. In a systematic review, articles reporting NBS leucine concentrations of confirmed patients were included. Our data showed that NBS of 1 963 465 newborns identified 4 MSUD patients and led to 118 false‐positive referrals (PPV 3.28%; incidence 1:491 000 newborns). In literature, leucine is the preferred NBS parameter. Total leucine (Xle) concentrations (mass‐spectrometry) of 53 detected and 8 false‐negative patients (sampling age within 25 hours in 3 patients) reported in literature ranged from 288 to 3376 (median 900) and 42 to 325 (median 209) μmol/L blood respectively. CLIR showed increasing Xle concentrations with sampling age and early NBS sampling and milder variant MSUD phenotypes with (nearly) normal biochemical profiles are causes of false‐negative NBS results. We evaluated the effect of additional screening markers and established the Xle/phenylalanine ratio as a promising additional marker ratio for increasing the PPV, while maintaining high sensitivity in the Dutch MSUD NBS.

**SYNOPSIS**

Screening of 1 963 465 infants for maple syrup urine disease (screening >72 hours, cut‐off value total leucine and valine ≥400 μmol/L blood) identified 4 patients and caused 118 false‐positive referrals (positive predictive value 3.28%). The total leucine/phenylalanine ratio is a promising additional marker ratio for increasing the positive predictive value, while maintaining sensitivity in the Dutch NBS program.

## 1 INTRODUCTION

Maple syrup urine disease (MSUD, MIM ID 248600) is a rare autosomal recessively inherited inborn error of metabolism caused by deficiency of the branched‐chain alpha‐keto acid dehydrogenase (BCKD; E.C.1.2.4.4) complex. Deficient BCKD complex activity leads to the accumulation of the branched‐chain amino acids (BCAAs): leucine (Leu), valine (Val), and isoleucine (Ile) of which Leu has acute neurotoxic effects. Based on the clinical presentation MSUD can be divided into five phenotypes: a classic phenotype, three milder phenotypic variants (intermediate, intermittent, and thiamine‐responsive), and a fifth phenotype caused by dihydrolipoyl dehydrogenase (E3)‐deficiency.^1^ Patients with the classic phenotype of MSUD have less than 3% residual BCKD complex activity and a clinical onset typically in the first weeks of life. Patients may present with feeding problems, a maple syrup odor, seizures, coma, and death.^2^ Patients with the variant phenotypes of MSUD have a higher residual enzyme activity and may have normal Leu values in anabolic state. Under severe catabolic stress, these patients can experience severe metabolic decompensations with increased Leu concentrations.^1^

The most important determinants of long‐term outcome in MSUD patients are the age at diagnosis and adequate metabolic control.^3^ MSUD is included in many newborn screening (NBS) programs which may enable detection before the occurrence of severe symptoms.^4^ Treatment with dietary Leu, Ile, and Val restriction, subsequent supplementation of Ile and Val, an emergency regime and close monitoring of Leu levels, improve the neurological and intellectual outcome of MSUD patients.^5^, ^6^, ^7^, ^8^ The introduction of tandem mass‐spectrometry (MSMS) for measurement of BCAAs in dried blood spots (DBS) markedly improved sensitivity and specificity.^9^, ^10^, ^11^, ^12^ In most NBS programs, MSMS determination of BCAAs does not differentiate between the isobaric amino acids Leu, Ile, allo‐isoleucine (Allo‐Ile), and hydroxyproline, providing a summation estimate of these compounds, termed “total Leu” (Xle) concentration.^9^, ^13^, ^14^ False‐positive NBS results may be caused by abnormal amino acid concentrations due to total parenteral nutrition (TPN), dietary causes, liver disease or by hydroxyprolinemia (MIM ID 237000), a benign biochemical condition.^15^

In the Netherlands, NBS for MSUD was added to the national screening program in 2007. From 2007 to 2017 primary screening parameters were Xle and Val concentrations measured by MSMS (cut‐off value [COV] of ≥400 μmol/L blood for both). After 11 years of NBS, we aimed to evaluate the effectiveness of the MSUD screening in the Dutch NBS program. We compared our results with data from a systematic literature review and consulted the Collaborative Laboratory Integrated Reports (CLIR) to explore strategies to optimize the neonatal screening for MSUD in the Netherlands.

## 2 METHODS

### 2.1 Evaluation of the Dutch NBS program for MSUD

#### 2.1.1 Study population

Anonymized data of all newborns screened from 2007 to 2017 were included in this study.

#### 2.1.2 Sample collection and analysis

For NBS in the Netherlands, DBS on filter paper are collected between 72 and 168 hours after birth. Five regional screening laboratories perform all NBS tests. From January 1, 2007 until January 10, 2008 the Neogram MSMS kit (Perkin Elmer, Turku, Finland) was used for the quantitative determination of amino acids on a Waters Micro tandem MS instrument (Waters, Milford, MA). From January 10, 2008 until December 31, 2017, the NeoBase Non‐derivatized MSMS kit (Perkin Elmer, Turku, Finland) was used for the measurement and evaluation of amino acids on the same instrument. These kits were comparable with regard to the concentrations measured. The upper limit of detection for Xle is 1200 μmol/L blood, and 700 μmol/L blood for Val. Newborns with a positive screening result (elevation of both Xle and Val required [COV ≥ 400 μmol/L blood]) were referred to a metabolic center for further diagnostics.

#### 2.1.3 Study outcomes

Xle and Val concentrations of the newborns diagnosed with MSUD were compared to the analytical data of all newborns collected by the Dutch NBS laboratories. To gain insight into the effectiveness of the Dutch program, the diagnostic accuracy in terms of positive and negative predictive value (PPV and NPV) and specificity and sensitivity was calculated. Because information on TPN at NBS sampling was not available, as an alternative the location of NBS sampling (home/hospital) of all false‐positive referrals was investigated. To improve our screening algorithm, CLIR was consulted to identify additional screening markers. In the Dutch NBS data, median, percentiles, and the hypothetical number of referrals based on several COVs were calculated for each marker using Microsoft Excel 2016. Also, of all MSUD false‐positive referrals from the 2007 to 2017 NBS cohort, the values of additional markers were extracted from the database.

##### Collaborative Laboratory Integrated Reports

CLIR (https://clir.mayo.edu) is a web application that maintains an interactive database of laboratory results from multiple sites. In addition to the database, CLIR provides a multivariate pattern recognition software and an interactive web tool, to allow post analytical interpretation of laboratory results.^16^, ^17^, ^18^ CLIR datasets comprise analytical data, age at heel prick collection, birth weight, gestational age and sex. The CLIR Productivity Tools are web‐based tools, which enable user utilization of the data in the database. The Productivity Tools were used to generate a “plot by condition” chart via the Productivity Tools menu: “plot by condition” (“AA disorder” MSUD); markers and marker ratios relevant to this study were selected. Unadjusted values were plotted as multiple of reference median vs markers. The “plot by condition” chart was used as a reference for informative markers in the evaluation of MSUD screening parameters. Furthermore the Productivity Tools were used to generate a “marker vs covariate plot” to assess the influence of age on concentrations measured.

### 2.2 Systematic review

#### 2.2.1 Research question

Primary outcomes of this systematic review were Leu and Val concentrations in MSUD patients detected by NBS worldwide, age at time of NBS, and the diagnosis and phenotype as defined by the authors. Secondary outcomes were the NBS algorithms and COVs applied, as well as the analytical methods used.

#### 2.2.2 Search strategy and study eligibility

A search strategy was developed and a literature search was conducted in OVID MEDLINE and OVID EMBASE from inception to November 5, 2019 to find studies on MSUD. Web of Science was used for cross‐checking of references. Retrieved records were imported and de‐duplicated in ENDNOTE X8. Complete search strategies are provided in Tables S1 and S2). Two authors (A. M. B. and K. S.) independently screened all titles and abstracts, and—if applicable—subsequently the full‐text version for eligibility. Considerations regarding discrepancies were discussed until consensus on eligibility was reached.

#### 2.2.3 Inclusion and exclusion criteria

We aimed to include all articles reporting on MSUD patients diagnosed or missed by NBS, with specification of Leu and/or Val concentrations and the age at the time of NBS. Only reports of MSUD patients detected by NBS performed in the first 2 weeks of life, before initiation of dietary treatment, and followed by clinical confirmation of the diagnosis, were included.

#### 2.2.4 Data extraction and analyses

Data extraction was performed separately by two researchers (A. M. B. and K. S.) using the predefined criteria. Because the origin of all data acquired for the systematic review was too heterogeneous for meta‐analysis, our results are presented solely in a descriptive manner, divided per publication. Descriptive statistics are presented with a median and (interquartile) range (IQR), appropriate for the distribution of the data. Literature data was analyzed using GraphPad PRISM 8.

## 3 RESULTS

### 3.1 Evaluation of the Dutch NBS program for MSUD

#### 3.1.1 NBS characteristics

From January 1, 2007 to December 31, 2017, MSUD NBS was performed for 1 963 465 newborns in the Netherlands. A total of 122 newborns were positive in the NBS analysis (Xle and Val concentrations ≥400 μmol/L blood) and referred to a metabolic center. During this 11‐year period, 4 newborns were diagnosed with MSUD and 118 were found to be false‐positive. Of these 118, sampling for NBS was performed at home in 65 newborns, in the hospital in 52 newborns (hospital admission after age 72 hours indicating a health issue) and for one the location of sample collection was unknown. TPN may have been the cause of the abnormal NBS results for a significant number of the false positives admitted in hospital at time of NBS sampling. No false‐negative results have been reported in the Dutch Diagnosis Registration Metabolic Diseases (DDRMD, https://www.ddrmd.nl/) to date. Because all newly diagnosed patients with a metabolic disorder are seen in a metabolic center and registered by the treating physician in the DDRMD, this leads to a probable sensitivity of 100%, a specificity of 99.994%, a probable NPV of 100%, and a PPV of 3.28%. The incidence of MSUD in the Netherlands during the eleven years of this study was 1:491 000 newborns. Xle and Val year medians and percentiles were comparable over the years (Table S3).

#### 3.1.2 Xle and Val concentrations of referred newborns

The four patients diagnosed with MSUD via NBS had Xle and Val concentrations of >1200 and 543, >1200 and 467, 947 and 548, and 697 and 430 μmol/L blood, respectively. The false‐positive referrals had a median Xle of 451 μmol/L blood (IQR 420‐502, range 400 to >1200) and median Val of 449 μmol/L blood (IQR 416‐502, range 400 to >700) (Table 1).

**TABLE 1:** Values of informative screening markers and ratios (median and percentiles) of 1 963 461 newborns screened for MSUD (including false‐positive referrals), false‐positive referrals (n = 118), and MSUD patients (n = 4)

2007‐2017 screening	Screened newborns (n = 1 963 461)^a^			False‐positives (n = 118)		MSUD patients (n = 4)			
Markers and ratios	Median	99.9th perc	99.99th perc	Median	95th perc	Patient 1	Patient 2	Patient 3	Patient 4
Xle	159	372	450	451	838	>1200	>1200	947	697
Val	129	354	443	449	643	543	467	548	430
(Xle + Val)/(Phe + Tyr)	2.04	4.43	5.97	3.06	6.34	>17.09	>18.32	11.59	6.55
Xle/Tyr	1.83	8.10	19.50	2.45	11.11	>27.91	>22.64	13.15	6.28
Xle/Phe	2.96	5.82	6.71	3.82	5.64	>20.34	>31.58	16.61	11.43
Val/Phe	2.41	5.41	6.38	3.99	5.86	9.2	12.29	9.61	7.05

*Note*: Xle and Val concentrations and cut‐off values in μmol/L blood.

Abbreviations: MSUD, maple syrup urine disease; Phe, phenylalanine; Tyr, tyrosine; Val, valine; Xle, total leucine.

a Excluding four true‐positive referrals.

#### 3.1.3 Influence of sampling age on Xle concentrations

**FIGURE 1:**
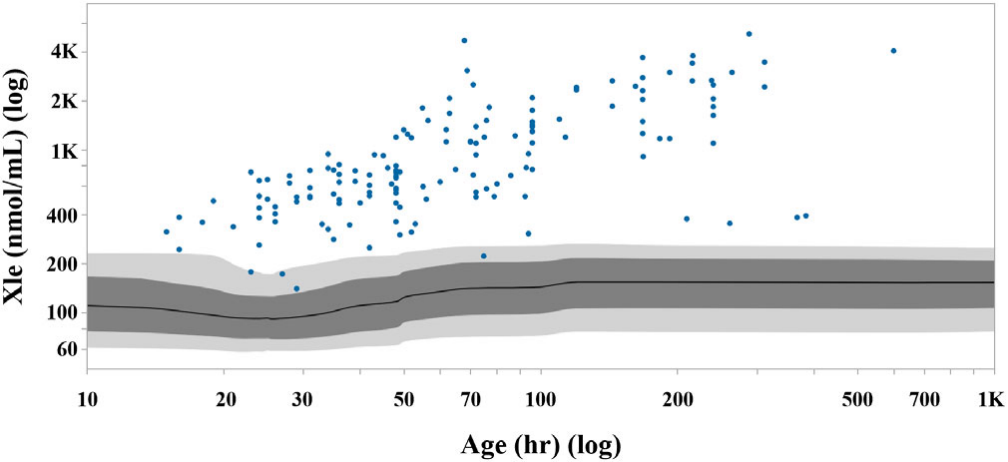
CLIR marker vs covariate plot showing the distribution of Xle concentrations (nmol/mL) in confirmed MSUD cases (n = 154) from the CLIR database with covariate “age at heel prick” (hours). Individual MSUD cases are represented by circles. Covariate values plotted on log scale. Covariate adjusted moving percentiles of the reference population (n = 2 210 428) are depicted in grey (1–99 percentile, 10‐90 percentile, and 50 percentile ranges). The NBS sample was obtained at ≤72 hours for 95 MSUD cases and >73 hours for 59 MSUD cases. CLIR, Collaborative Laboratory Integrated Reports; MSUD, maple syrup urine disease; NBS, newborn screening; Xle, isoleucine + leucine

The CLIR Productivity Tools showed that Xle concentrations increase with NBS sampling age (Figure 1).

#### 3.1.4 Identification of informative screening markers for MSUD via the CLIR tools and diagnostic accuracy of markers and ratios

The CLIR Productivity Tools were consulted to detect discriminative screening markers and ratios. Considering only markers which are included in the Dutch NBS, the following marker ratios were identified: Xle/tyrosine (Tyr), Xle/phenylalanine (Phe), (Xle + Val)/(Phe + Tyr), Xle/Tyr, Val/Phe, and C5‐carnitine (C5)/Xle. C5 concentrations in the Dutch NBS are reported with one decimal and were 0.1 μmol/L blood in the majority of our screened population, and also in all four MSUD patients. Therefore, a discriminative COV for the C5/Xle ratio is predominantly determined by the Xle concentration, and is not of added value. Figure S1 shows the relevant discriminative ratios as well as Allo‐Ile, which is a pathognomonic marker used as second tier but not currently included in Dutch NBS as a separate marker. We reevaluated the Dutch screening data (complete cohort, confirmed patients, false‐positive referrals 2007‐2017) using the marker ratios reported in CLIR to be discriminative (Table 1). Subsequently, we calculated hypothetical referral numbers and PPVs in our Dutch NBS cohort by using different COVs that would maintain 100% sensitivity (under the assumption no patients were missed in our NBS). The most promising marker ratio was Xle/Phe. When used as a first‐tier test, the Xle/Phe ratio PPVs were 14.3%, 30.8%, and 36.4% with COVs of ≥8, ≥9, and ≥10 respectively. Furthermore, we calculated the Xle/Phe ratio of the false‐positive referrals from the 2007 to 2017 NBS cohort, where the ratio proved discriminative with lower COVs (Table 2).

**TABLE 2:** Additional markers and ratios and the hypothetical effect of several cut‐off values, maintaining a 100% sensitivity, on referral numbers, and the number of 2007‐2017 false‐positive referrals above the respective cut‐off value

2007‐2017 screening	Cut‐off value	True‐positives	False‐positives	PPV
Xle + Val	≥400 + ≥400	4	118	3.28%

					2007‐2017 false‐positives above cut‐off value	
Markers and ratios	Cut‐off value	True‐positives	Referrals (n)^a^	PPV^b^	n	%
Xle	≥400	4	724	0.55%		
Val	≥400	4	565	0.70%		
(Xle + Val)/(Phe + Tyr)	≥4	4	4048	0.10%	24	20.34%
	≥5	4	864	0.46%	16	13.56%
	≥6	4	191	2.05%	7	5.93%
Xle/Tyr	≥4	4	16 628	0.02%	32	27.12%
	≥5	4	7007	0.06%	27	22.88%
	≥6	4	4226	0.10%	23	19.49%
Xle/Phe	≥4	4	177 188	0.00%	50	42.37%
	≥4.5	4	58 413	0.01%	25	21.19%
	≥5	4	17 249	0.02%	17	14.41%
	≥6	4	1233	0.32%	5	4.24%
	≥7	4	108	3.57%	2	1.70%
	≥8	4	24	14.29%	1	0.85%
	≥9	4	9	30.77%	1	0.85%
	≥10	4	7	36.36%	1	0.85%
Val/Phe	≥4	4	54 826	0.01%	59	50.00%
	≥5	4	5413	0.07%	24	20.34%
	≥6	4	464	0.86%	6	5.08%
	≥7	4	47	7.84%	1	0.85%

*Note*: Xle and Val concentrations and cut‐off values in μmol/L blood.

Abbreviations: Phe, phenylalanine; PPV, positive predictive value; Tyr, tyrosine; Val, valine; Xle, total leucine.

a Hypothetical referral number excluding the four true‐positive referrals.

b Calculated PPV under the assumption of zero false‐negative referrals, maintaining 100% sensitivity.

### 3.2 Systematic review

#### 3.2.1 Study eligibility

The literature search identified 2250 unique publications and screening of titles and abstracts, full‐texts and references resulted in the inclusion of 18 articles (Figure S2).

#### 3.2.2 Inclusion

The 18 articles included, reported on 112 screened MSUD patients: 104 diagnosed and 8 false‐negative referrals.^4^, ^5^, ^8^, ^9^, ^12^, ^13^, ^19^, ^20^, ^21^, ^22^, ^23^, ^24^, ^25^, ^26^, ^27^, ^28^, ^29^, ^30^

#### 3.2.3 Screening methods

NBS results from MSMS methods were commonly reported as Xle or combined Leu and Ile concentrations. MSMS was reported as the screening method in 13 articles describing 53 detected MSUD patients and 8 false‐negative referrals by NBS (Table 3). Five articles reported 51 MSUD patients detected by other methods (Table S4).^12^, ^21^, ^24^, ^27^, ^28^

**TABLE 3:** Systematic review of the literature: newborn screening results by MSMS

Author (country)	Period	Screening method	Case #	Age at NBS	Xle (μmol/L blood)	COV (μmol/L blood)	Val (μmol/L blood)	COV (μmol/L blood)	Diagnosis as defined by authors
MSUD patients detected by NBS									
Agadi et al^19^ (USA)		MSMS^a^	#1	First 8 days	739	362	317	311	MSUD
Bhattacharya et al^20^ (Australia)	1998‐2005	ES‐TMS	#2	3	>1000	400	—	—	MSUD: “Classical”
			#3	3	>1200				MSUD: “Classical”
			#4	3	>1500				MSUD: “Classical”
Chace et al^9^ (USA)		MSMS	#5	24 h	765	—	174	—	MSUD
			#6	24 h	748		404		MSUD
			#7	24 h	756		186		MSUD
			#8	24 h	647		359		MSUD
			#9	24 h	569		404		MSUD
			#10	24 h	1019		1162		MSUD
			#11^b^	17 h	344		303		MSUD
Couce et al^5^ (Spain)	2001‐2013	TMS	#12	7	1467	380	—	—	MSUD: “Moderate”
			#13	7	3376				MSUD: “Classical”
			#14	2	590				MSUD: “Classical”
			#15	3	514				MSUD: “Moderate”
			#16	3	1124				MSUD: “Classical”
			#17	8	565				MSUD: “Classical”
			#18	3	1141				MSUD: “Moderate”
Fingerhut et al^13^ (Germany and Austria)	1999‐2005	ESI‐MSMS	#19	3	835	275‐393	830	—	MSUD: “Variant”
			#20	8	1030		—		MSUD: “Variant”
			#21	3	485		—		MSUD: “Variant”
			#22	4	405		427		MSUD: “Variant”
			#23	3	483		469		MSUD: “Variant”
			#24	4	288		261		MSUD: “Variant”
			#25	3	298		249		MSUD: “Variant”
			#26	4	642		363		MSUD: “Variant”
			#27	4	586		476		MSUD: “Variant”
			#28	3	1039		479		MSUD: “Classical”
			#29	4	1450		—		MSUD: “Classical”
			#30	4	900		—		MSUD: “Classical”
			#31	4	1080		—		MSUD: “Classical”
			#32	4	524		620		MSUD: “Classical”
			#33	5	2183		823		MSUD: “Classical”
			#34	2	1188		—		MSUD: “Classical”
			#35	3	1400		—		MSUD: “Classical”
			#36	4	2068		—		MSUD: “Classical”
			#37	3	572		538		MSUD: “Classical”
Hassan et al^22^ (Egypt)	2008	ESI‐MSMS	#38	3–7	1945	290	497	270	MSUD
Heldt et al^4^ (Germany)	2002‐2005	TMS	#39	3	1076	229	—	—	MSUD: “Classic”
			#40	3	633				MSUD: “Classic”
Huang et al^23^ (Taiwan)	2001‐2004	MSMS	#41	First 2 weeks	1850	171	—	—	MSUD
Myers et al^25^ (Canada)		^a^	#42	2	373	250	—	—	MSUD
Simon et al^8^ (Germany and Austria)	1999‐2005	MSMS	#43	3	1039	390	—	—	MSUD” “Classical”
			#44	4	1450				MSUD: “Classical”
			#45	4	900				MSUD: ”Classical”
			#46	4	1080				MSUD: “Classical”
			#47	4	524				MSUD: “Classical”
			#48	5	2183				MSUD: “Classical”
			#49	2	1188				MSUD: “Classical”
			#50	3	1400				MSUD: “Classical”
			#51	4	2068				MSUD: “Classical”
			#52	3	572				MSUD: “Classical”
Zytkovicz et al^30^ (USA)	1999‐2001	MSMS	#53	2	458	373 (re‐test)	—	—	MSUD
MSUD patients missed by NBS									
Bhattacharya et al^20^ (Australia)	1998‐2005	ES‐TMS	#54	48‐72 h	325	400	—	—	MSUD: “Intermittent”
			#55	48‐72 h	209				MSUD: “Intermittent”
Yunus et al^29^ (Malaysia)		MSMS	#56	First 7 days	250‐300	300 → 250	—	—	MSUD
Puckett et al^26^ (USA)	2005‐2009	MSMS	#57	4	295	200^c^	216	—	MSUD “Variant”
			#58	39 h	242		232		MSUD: “Variant”
			#59	25 h	42		50		MSUD: “Variant”
			#60	12 h	67		66		MSUD: “Variant”
			#61	19 h	160		185		MSUD: “Variant”

Abbreviations: COV, cut‐off value; ES, electrospray; ESI, electrospray ionization; h, hour; MSMS or TMS, tandem mass spectrometry; MSUD, maple syrup urine disease; NBS, newborn screening; Val, valine; Xle, total leucine.

a Likely MS/MS, but not reported as such.

b Identified as MSUD by Xle/phenylalanine ratio of 9.0.

c Xle ≥200 and a Xle/alanine ratio ≥ 1.5 required for referral.

#### 3.2.4 Screening strategy and COVs

Chace et al^9^ first described the use of MSMS for MSUD NBS and recommended referral based on Xle in combination with a Xle/Phe ratio for an improved detection. In subsequent studies, referral for MSUD was based on an elevated Xle or Leu.^4^, ^5^, ^8^, ^12^, ^13^, ^20^, ^21^, ^23^, ^25^, ^27^, ^28^, ^29^, ^30^ Some required Val for referral,^19^, ^22^, ^24^, ^27^ while others did report Val, but without COV.^9^, ^13^, ^26^ One program included a Xle/alanine (Ala) ratio.^26^ COVs were individually established in each NBS program, commonly based on experience from a pilot study or from experiences in other centers. COVs of Xle determined by MSMS ranged from 171 to 400 μmol/L blood (Table 3). Val COVs were often unreported (Tables 3 and S4).

#### 3.2.5 NBS for MSUD by MSMS

##### Xle and Val concentrations measured in MSUD patients at NBS

Fifty‐two out of 53 NBS detected MSUD patients reported in literature were identified by MSMS with an elevated Xle and Val or an elevated Xle alone. The Xle concentration in this group ranged from 288 to 3376 μmol/L blood (median 900) (Figure S3).^4^, ^5^, ^8^, ^9^, ^13^, ^19^, ^20^, ^22^, ^23^, ^25^, ^26^, ^29^, ^30^ Twenty‐nine patients were classified as classic MSUD and had Xle concentrations ranging from 524 to 3376 μmol/L blood (median 1080). Twelve patients were classified as variant MSUD (three reported as moderate and nine unspecified by authors), with Xle concentrations ranging from 288 to 1467 μmol/L blood (median 550). The remaining 12 patients were unclassified by the authors (Table 3). In 20 MSUD patients NBS Val was determined and ranged from 174 to 1162 μmol/L blood (median 416). The median age at NBS of patients with available data was 3 days (range 17 hours to 8 days) (Table 3).

##### MSUD NBS false‐negative referrals

In literature eight patients were reported as false‐negative by NBS programs with Xle COVs of 400, 300, and 200 μmol/L blood respectively (Table 3). The program with a COV of 200 μmol/L blood for Xle also required an elevated Xle/Ala ratio for referral. Xle concentrations of seven of the eight false‐negative patients ranged from 42 to 325 μmol/L blood (median 209) (Table 3 and Figure S3). The Xle concentration of the eighth patient was not reported but the program decreased the COV from 300 to 250 μmol/L blood following this missed case. NBS sampling age of the false‐negative referrals varied from 12 hours to 4 days (median 2 days) and the lowest Xle concentrations (42‐160 μmol/L blood) were from samples taken in the first 25 hours of life (three out of eight). Of the eight false‐negative MSUD patients, two were classified by the authors as intermittent and five as variant MSUD.

##### Xle/Phe ratio in MSUD patients

No program reported using Xle/Phe ratio as primary screening parameter, but data on the ratio in patients with NBS sampling age > 72 hours was provided for seven patients in two articles. In two MSUD patients classified as classical (Xle 524 and 2183 μmol/L blood) the Xle/Phe ratios were 7.5 and 28.6,^13^ and in five variant MSUD patients (Xle 295, 405, 288, 586, and 642 μmol/L blood) the Xle/Phe ratios were 4.1, 4.4, 4.5, 7.2, and 13.2 respectively.^13^, ^26^

##### The role of Val in MSMS strategies

Few Val concentrations were reported. Remarkably, in four reported patients (three unclassified and one variant MSUD), Val at NBS (174, 186, 359, 363 μmol/L blood) was well below the COV in the Netherlands, while Xle was above 400 μmol/L blood (Table 3). Importantly, the age at NBS was 24 hours in three patients and 4 days in the other patient.

#### 3.2.6 MSUD patients screened by other NBS methods

Amino acid concentrations in 51 patients detected by other methods are reported in Table S4. No reports of patients missed by these methods were found.

## 4 DISCUSSION

Our study shows that in 11 years of MSUD NBS four patients were identified and no false‐negative referrals for MSUD were reported, suggesting an excellent sensitivity of the Dutch 2007 to 2017 NBS. This results in an incidence of MSUD in the Dutch population of 1:491 000 newborns. The specificity of our NBS algorithm (both Xle and Val ≥400 μmol/L blood) was 99.994%, with a PPV of 3.28%. We explored possibilities to improve the PPV and showed that the use of the Xle/Phe ratio may be a promising strategy. Finally, we performed a systematic review of the literature. In literature, the preferred strategy for MSUD NBS is using Xle (or Leu) as single identification parameter, in some studies combined with Val. The lowest Xle concentration in the four confirmed MSUD patients in the Netherlands was 697 μmol/L blood, and in literature all classic MSUD patients screened by MSMS had Xle concentrations of 524 μmol/L blood or higher, which is above our Xle COV. Importantly, there is a large overlap in Xle concentrations between variant and classic patients. The literature search clearly demonstrated that patients with variant MSUD may even have normal Xle (and Val) concentrations at time of sampling. The lowest Xle concentration in reported variant MSUD patients, with an age at NBS ≥3 days, was 288 μmol/L blood. Moreover, seven variant patients with normal Xle concentrations were missed by NBS. Thus, patients with a phenotypic variant MSUD may be at risk for a false‐negative NBS result, due to their variable biochemical profile at time of NBS. Importantly, three of these variant patients were screened within 25 hours after birth. Since CLIR clearly demonstrates a strong effect of sampling age on Xle concentrations in MSUD patients (Figure 1), early NBS blood sampling can also be a factor contributing to these false‐negative results.

While it is important to detect as many variant patients as possible to prevent the severe metabolic decompensation that may occur at times of catabolism,^1^ this should not result in a higher number of false‐positive referrals and normal biochemical profiles of these patients at the time of NBS will hamper detection.

The PPV of our NBS program (2007‐2017) for MSUD was relatively low (3.28%). Contributors to the high number of false‐positive referrals may include liver disease, TPN (possibly in 52 newborns admitted to the hospital at the time of NBS), and the fact that the MSMS system used measures Xle concentrations, including other isobaric compounds. In literature measures to reduce false‐positive referrals due to TPN have been proposed,^31^ which are currently not implemented in the Dutch NBS protocols.

To improve specificity, several publications proposed Allo‐Ile as a highly valuable second‐tier test.^14^, ^24^, ^32^, ^33^, ^34^ Indeed, in some studies Allo‐Ile was demonstrated to be undetectable in healthy neonates and elevated in all classical MSUD cases (^14^, ^35^); however, in variant MSUD undetectable Allo‐Ile concentrations have been reported.^26^ Nevertheless, data from the CLIR database demonstrated that Allo‐Ile clearly distinguishes healthy neonates from MSUD patients (Figure S1). However, no information in CLIR is available concerning severity of the MSUD phenotype (classic or variant). Thus, two important causes of false‐negative NBS Xle results have to be considered: early NBS blood sampling and the mild phenotypic variant MSUD cases. As NBS blood sampling in the Netherlands occurs from the age of 72 hours, the effect of age will be negligible in our NBS program. Because variant MSUD patients could have a normal biochemical profile at time of NBS, it cannot be excluded that these patients may still be missed.

In the present reevaluation of the Dutch NBS population data (2007‐2017), we selected the Xle/Phe ratio from the CLIR database. As a first‐tier test this ratio was promising in increasing the PPV in our cohort (Table 2). Taking into account that the lowest Xle/Phe ratio of our four patients was 11.43, a Xle/Phe ratio COV ≥8 could maintain sensitivity while increasing the PPV to 14.3%. However, the literature demonstrated that the predictive value of the Xle/Phe ratio for variant MSUD is less clear, as most variant patients screened after 72 hours of age were reported to have Xle/Phe ratios below 8, and even as low as 4.1.^13^, ^26^ Alternatively, adding the Xle/Phe ratio with a COV of 4 or 4.5 to the 2007 to 2017 NBS strategy could maintain current sensitivity, while preventing 57.63% and 78.81% of false‐positive referrals respectively. To improve the detection of variant MUSD patients the possibility of lowering the Xle and Val COVs in combination with the Xle/Phe ratio could be further explored.

A limitation of our literature study is that a clear classification of the MSUD phenotype was often lacking. This, combined with the rarity of the disease, provides only limited insight into the biochemical profiles of the variant MSUD patients and its consequences for early detection in NBS programs worldwide. The reported amino acid concentrations were measured by different assays in a 25‐year period of MSMS development, which may hamper reliable comparison of NBS results between studies and with our MSMS approach. As only four MSUD patients are known in the Netherlands from NBS, no long‐term conclusions can be made regarding the sensitivity of an alternative NBS strategy. Lastly, a limitation of both the Dutch NBS data and the systematic review is that no information regarding the time of blood sampling in relation to the feeding times is known. This may have been of influence on the BCAA concentrations measured.

In conclusion, 11 years of NBS for MSUD in the Netherlands has led to the detection of 4 MSUD patients, at the cost of 118 false‐positive referrals. We evaluated the effect of additional screening markers and established the Xle/Phe ratio as a promising additional marker ratio to our MSUD NBS strategy and implementation in the Dutch NBS program should be considered. The Dutch NBS program is the responsibility of the Dutch Screening Program Management assisted by a clinical expert advisory board and ultimately the Program Committee for NBS.

## CONFLICT OF INTEREST

A. M. B. has received a speakers fee from Nutricia and has been a member of advisory boards for Biomarin. F. S. is a member of advisory boards, has obtained grants from or performed consultancy for Agios, Applied Pharma Research, Arla Food Int., Eurocept, BioMarin, Lucane, Nestle‐Codexis Alliance, Nutricia, Orphan Europe, Origin, Biosciences, Rivium Medical BV, Sobi and Vivet, Alexion, NPKUA, Pluvia, Biotech and MendeliKABS. For all these activities the UMCG received a fee. All other authors have nothing to declare.

## AUTHOR CONTRIBUTIONS

Kevin Stroek, Anita Boelen, Evelien Kemper, and Annet Bosch were involved in conception and design, acquisition, analysis and interpretation of data, and drafting of the manuscript. Marelle Bouva and Rose Maase were involved in acquisition, analysis, and interpretation of data, and critical revision of the manuscript. Peter Schielen was involved in conception, acquisition, analysis and interpretation of data, and critical revision of the manuscript. Henk Engel and Bernadette Jakobs were involved in conception, acquisition of data, and critical revision of the manuscript. Margot Mulder, M. E. Rubio‐Gozalbo, Francjan van Spronsen, Gepke Visser, Maaike de Vries, Monique de Sain‐van der Velden, Leo Kluijtmans, and Monique Williams were involved in conception, interpretation of data, and critical revision of the manuscript. Annemieke Heijboer was involved in interpretation of data and critical revision of the manuscript. All authors have read and approved the final manuscript.

## Supporting information


**TABLE S1** MEDLINE search strategy
**TABLE S2** Embase search strategy
**TABLE S3** Total leucine and valine measurements in the screened population per year of NBS in the Netherlands
**TABLE S4** Systematic review of the literature: newborn screening results by other methods

**FIGURE S1** CLIR “Plot by Condition” chart showing disease ranges (1%, 10%, 50%, 90%, and 99%iles, respectively) in neonatal blood spots of patients with MSUD (N = 275). The reference population (corresponding to the 1‐99%ile range; Xle N = 2.45 million; Val N = 2.31 million; AlloIle N = 635; Val/Phe N = 2.18 million; Xle/Phe N = 2.39 million; Xle/Tyr N = 2.26 million; (Xle + Val)/(Phe + Tyr) N = 1.92 million) is depicted in green. Abbreviations as follows: Allo‐Ile: allo‐isoleucine, CLIR: Collaborative Laboratory Integrated Reports, MSUD: Maple Syrup Urine Disease, Phe: phenylalanine, Tyr: tyrosine, Val: valine, Xle: isoleucine + leucine

**FIGURE S2** Flowchart with the selection process of the systematic review

**FIGURE S3** Total leucine (Xle) concentrations (median and IQR) measured in MSMS NBS in MSUD patients, including both true‐positive and false‐negative literature results
