# Supplementary material for: Evaluation of 11 years of newborn screening for maple syrup urine disease in the Netherlands and a systematic review of the literature: Strategies for optimization
Source: JIMD Rep. 2020 May 13;54(1):68–78. doi: 10.1002/jmd2.12124 (PMC7358668; doi:10.1002/jmd2.12124)
Supplement: Supplementary file 1 — TABLE S1 MEDLINE search strategy TABLE S2 Embase search strategy TABLE S3 Total leucine and valine measurements in the screened population per year of NBS in the Netherlands TABLE S4 Systematic review of the literature: newborn screening results by other methods [file JMD2-54-68-s002.docx]

**Supplementary materials**

**Table S1** MEDLINE search strategy
Database(s): Ovid MEDLINE(R) Epub Ahead of Print, In-Process & Other Non-Indexed Citations, Ovid MEDLINE(R) Daily and Ovid MEDLINE(R) 1946 to Present 
Search Strategy: 2019-11-05

| **#** | **Searches** | **Results** |
| --- | --- | --- |
| 1 | maple syrup urine disease/ | 1127 |
| 2 | (maple syrup urin* adj (diseas* or disorder*)).tw,kf. | 1110 |
| 3 | (maple syrup adj (diseas* or disorder*)).tw,kf. | 41 |
| 4 | (CMSUD or classic MSUD).tw,kf. or (MSUD.tw,kf. and ((screen* and newborn*) or BCAA* or branched-chain or BCKDK or inborn error* or metabolic dis*).mp.) | 448 |
| 5 | ((BCKD or BCKDH or keto-acid-decarboxylas*) adj3 deficien*).tw,kf. | 13 |
| 6 | (branched-chain adj2 (amino acid?emi* or ketoaciduri* or keto-aciduri* or ((keto acid or ketoacyl or keto-acyl) adj dehydrogenase deficien*))).tw,kf. | 52 |
| 7 | leucinosis.tw,kf. | 42 |
| 8 | or/1-7 [ MSUD ] | 1470 |
| 9 | (exp animals/ not humans/) or (dog or dogs or canine or pig or pigs or piglet* or rodent* or rat or rats or murine or mouse or mice or drosophil* or yeast* or saccharomyc* or tetrahymen* or escherich* or bacteri* or cell line* or ((animal* or mammalian*) not human*)).ti. | 5412012 |
| 10 | 8 not 9 [ MSUD not in animals] | 1334 |
| 11 | remove duplicates from 10 [ MSUD not in animals -deduplicated ] | 1307 |

**Table S2** Embase search strategy
Database(s): Embase Classic+Embase 1947 to 2019 November 5 
Search Strategy: 2019-11-05

| **#** | **Searches** | **Results** |
| --- | --- | --- |
| 1 | maple syrup urine disease/ | 2269 |
| 2 | (maple syrup urin* adj (diseas* or disorder*)).tw,kw. | 1603 |
| 3 | (maple syrup adj (diseas* or disorder*)).tw,kw. | 108 |
| 4 | (CMSUD or classic MSUD).tw,kw. or (MSUD.tw,kw. and ((screen* and newborn*) or BCAA* or branched-chain or BCKDK or inborn error* or metabolic dis*).mp.) | 781 |
| 5 | ((BCKD or BCKDH or keto-acid-decarboxylas*) adj3 deficien*).tw,kw. | 16 |
| 6 | (branched-chain adj2 (amino acid?emi* or ketoaciduri* or keto-aciduri* or ((keto acid or ketoacyl or keto-acyl) adj dehydrogenase deficien*))).tw,kw. | 75 |
| 7 | leucinosis.tw,kw. | 69 |
| 8 | or/1-7 [ MSUD ] | 2525 |
| 9 | ((animal.hw. or nonhuman/) not human/) or (dog or dogs or canine or pig or pigs or piglet* or rodent* or rat or rats or murine or mouse or mice or drosophil* or yeast* or saccharomyc* or tetrahymen* or escherich* or bacteri* or cell line* or ((animal* or mammalian*) not human*)).ti. | 7220125 |
| 10 | 8 not 9 [ MSUD not in animals] | 2330 |
| 11 | remove duplicates from 10 [ MSUD not in animals -deduplicated ] | 2249 |
| 12 | 11 not medline.cr. | 1888 |

**Table S3** Total leucine and valine measurements in the screened population per year of NBS in the Netherlands

| Year |  |  |  | Total leucine (µmol/L blood) | | | | Valine (µmol/L blood) | | | |
| --- | --- | --- | --- | --- | --- | --- | --- | --- | --- | --- | --- |
|  | No. of first heel pricks | No. of true-positives | No. of false-positives | Median | 99.90% | 99.99% |  | Median | 99.90% | 99.99% |  |
| 2007 | 182443 | 1 | 10 | 150 | 365 | 457 |  | 120 | 292 | 370 |  |
| 2008^a^ | 186776 | 0 | 5 | 147 | 347 | 433 |  | 117 | 303 | 383 |  |
| 2009 | 185812 | 0 | 5 | 143 | 338 | 406 |  | 113 | 305 | 385 |  |
| 2010^b^ | 185288 | 1 | 18 | 166 | 383 | 470 |  | 140 | 382 | 466 |  |
| 2011 | 180584 | 0 | 13 | 169 | 382 | 454 |  | 136 | 344 | 431 |  |
| 2012 | 176859 | 0 | 4 | 161 | 369 | 455 |  | 119 | 306 | 374 |  |
| 2013 | 175331 | 0 | 6 | 164 | 372 | 450 |  | 135 | 334 | 410 |  |
| 2014 | 176067 | 0 | 10 | 160 | 360 | 437 |  | 129 | 329 | 398 |  |
| 2015 | 171445 | 1 | 11 | 163 | 380 | 446 |  | 142 | 384 | 459 |  |
| 2016 | 173052 | 1 | 19 | 160 | 372 | 459 |  | 136 | 371 | 466 |  |
| 2017 | 169808 | 0 | 17 | 166 | 389 | 452 |  | 138 | 394 | 482 |  |

^a^ Transition from NeoGram to NeoBase™ Non-derivatized MSMS kit in October 2008
^b^ Introduction of Ahlstrom blood spot paper in 2010

**Table S4** Systematic review of the literature: newborn screening results by other methods

| Author (Country) | Period | Method | Case # | Age at NBS | Leu* (µmol/L blood) | COV (µmol/L blood) | Val (µmol/L blood) | COV (µmol/L blood) | Diagnosis as defined by authors | | | |  |  |
| --- | --- | --- | --- | --- | --- | --- | --- | --- | --- | --- | --- | --- | --- | --- |
| de Castro-Hamoy et al 2017 (Phillipines) | 2012-2014 | Enzymatic/ Colorimetric testing, Alisei kit | #62-85 (N=24) | Mean 4 range [1-11] | Mean 1067 (SD 529), range [312-2330] | 300 (recall 700) | - | - | MSUD |  |  |  | |  |
| Jeong et al 2011 (South Korea) |  | HPLEC-UV | #86-88 (N=3) | First 7 days | Mean Leu 958 (429-1856);  Mean Ile  358 (101-668);  Mean Allo-Ile 238 (137-304) | Leu 230    Ile 105  Allo-Ile 5 | Mean 614 (401-940) | 480 | MSUD |  |  |  | |  |
| Naylor & Guthrie 1978 (USA & New Zealand) | 1965-1976 | BIA | #89  #90  #91  #92  #93  #94  #95  #96  #97  #98  #99 | 4  3  2  4  4  3  3  5  5  5  2 | 1525  915  457-610  457  1525  915  610  1525  1525  1525  610-762 | 305 | - | - | MSUD –‘’Classical’’  MSUD –‘’Classical’’  MSUD –‘’Intermediate’’  MSUD –‘’Intermediate’’  MSUD –‘’Classical’’  MSUD –‘’Classical’’  MSUD –‘’Intermediate’’  MSUD –‘’Classical’’  MSUD –‘’Classical’’  MSUD –‘’Classical’’  MSUD –‘’Classical’’ | | | | |  |
| Puliyanda et al 2002 (USA) |  | Ion Exchange Chromatography | #100 | First 2 weeks | Leu 3472  Ile 473  Allo-Ile 260 | Leu 153  Ile 105  Allo-Ile 0 | 819 | 300 | MSUD |  |  |  | |  |
| Tada et al 1984 (Japan) | 1977-1982 | BIA | #101  #102  #103  #104  #105  #106  #107  #108  #109  #110  #111  #112 | 5-7  5-7  5-7  5-7  5-7  5-7  5-7  5-7  5-7  5-7  5-7  5-7 | 1677  2058  1982  5489  1220  610  5489  3202  1220  >1525  2211  4269 | - | - | - | MSUD  MSUD  MSUD  MSUD  MSUD  MSUD  MSUD  MSUD  MSUD  MSUD  MSUD  MSUD |  |  |  | |  |

BIA: Bacterial inhibition assay, COV: Cut-off value, HPLEC-UV: High-performance ligand-exchange chromatography-UV, Ile: isoleucine, Leu: leucine, MSUD: Maple syrup urine disease, NBS: Newborn screening, Val: valine
*: Leucine, or as specified
