# Supplementary material for: Evaluation of 11 years of newborn screening for maple syrup urine disease in the Netherlands and a systematic review of the literature: Strategies for optimization
Source: JIMD Rep. 2020 May 13;54(1):68–78. doi: 10.1002/jmd2.12124 (PMC7358668; doi:10.1002/jmd2.12124)
Supplement: Supplementary file 2 — FIGURE S1 CLIR “Plot by Condition” chart showing disease ranges (1%, 10%, 50%, 90%, and 99%iles, respectively) in neonatal blood spots of patients with MSUD (N = 275). The reference population (corresponding to the 1‐99%ile range; Xle N = 2.45 million; Val N = 2.31 million; AlloIle N = 635; Val/Phe N = 2.18 million; Xle/Phe N = 2.39 million; Xle/Tyr N = 2.26 million; (Xle + Val)/(Phe + Tyr) N = 1.92 million) is depicted in green. Abbreviations as follows: Allo‐Ile: allo‐isoleucine, CLIR: Collaborative Laboratory Integrated Reports, MSUD: Maple Syrup Urine Disease, Phe: phenylalanine, Tyr: tyrosine, Val: valine, Xle: isoleucine + leucine [file JMD2-54-68-s001.pdf]

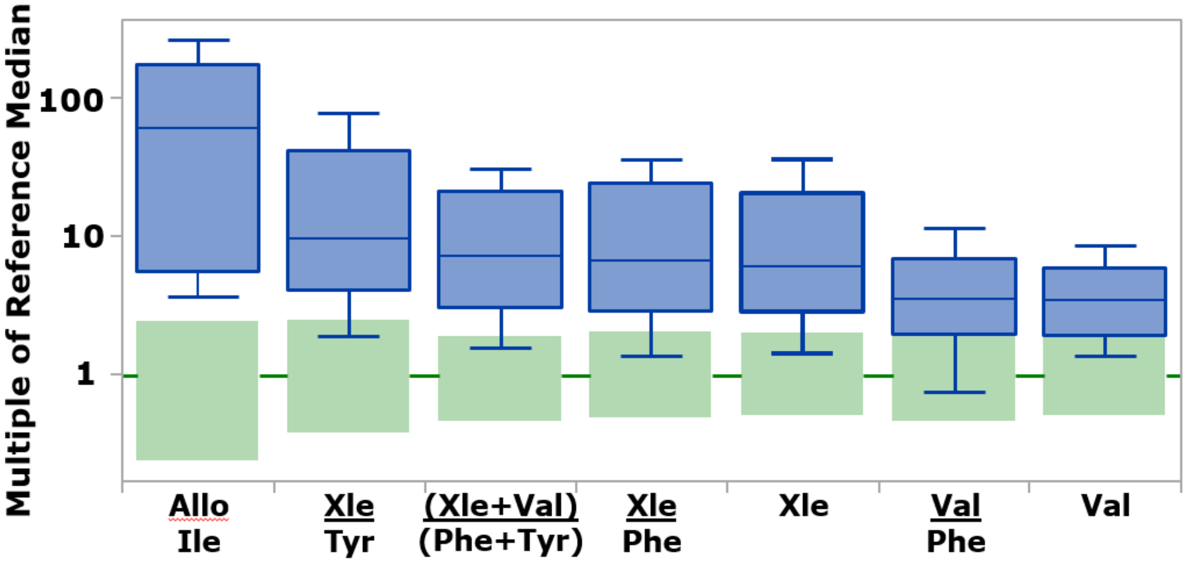

Figure S1 CLIR 'Plot by Condition' chart showing disease ranges (1%, 10%, 50%, 90%, and 99%iles, respectively) in neonatal blood spots of patients with MSUD (N=275).
The reference population (corresponding to the 1-99%ile range; Xle N=2.45 million; Val N=2.31 million; AlloIle N=635; Val/Phe N=2.18 million; Xle/Phe N=2.39 million; Xle/Tyr N=2.26 million; (Xle+Val)/(Phe+Tyr) N=1.92 million) is depicted in green. Abbreviations as follows: Allo-Ile: allo-isoleucine, CLIR: Collaborative Laboratory Integrated Reports, MSUD: Maple Syrup Urine Disease, Phe: phenylalanine, Tyr: tyrosine, Val: valine, Xle: isoleucine + leucine.
