# Supplementary figures and images for: Evaluation of 11 years of newborn screening for maple syrup urine disease in the Netherlands and a systematic review of the literature: Strategies for optimization
Source: JIMD Rep. 2020 May 13;54(1):68–78. doi: 10.1002/jmd2.12124 (PMC7358668; doi:10.1002/jmd2.12124)

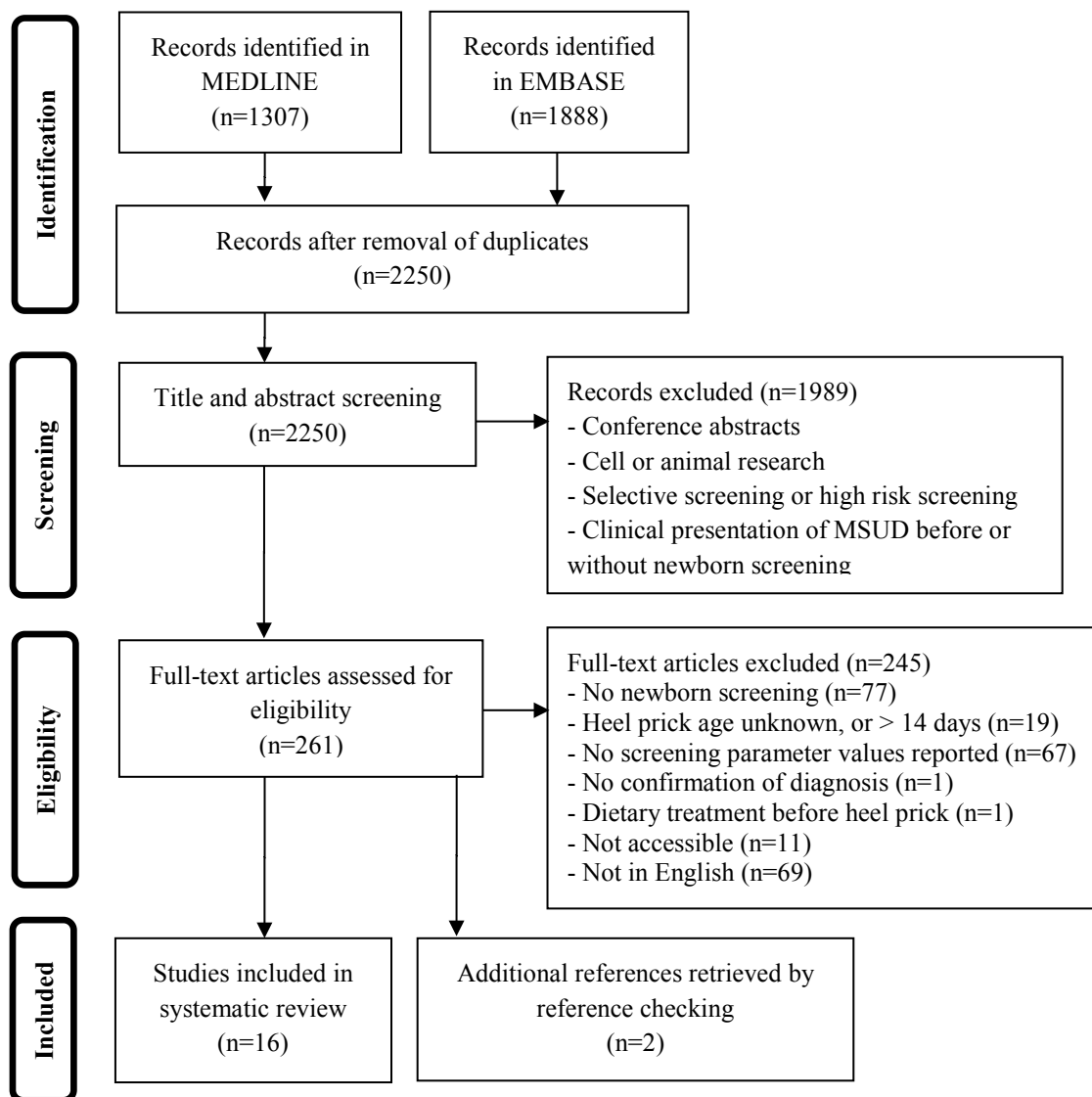

**Figure S2** Flowchart with the selection process of the systematic review

Supplement: Supplementary file 3 — FIGURE S2 Flowchart with the selection process of the systematic review [file JMD2-54-68-s003.pdf]
