# Supplementary material for: Evaluation of 11 years of newborn screening for maple syrup urine disease in the Netherlands and a systematic review of the literature: Strategies for optimization
Source: JIMD Rep. 2020 May 13;54(1):68–78. doi: 10.1002/jmd2.12124 (PMC7358668; doi:10.1002/jmd2.12124)
Supplement: Supplementary file 4 — FIGURE S3 Total leucine (Xle) concentrations (median and IQR) measured in MSMS NBS in MSUD patients, including both true‐positive and false‐negative literature results [file JMD2-54-68-s004.pdf]

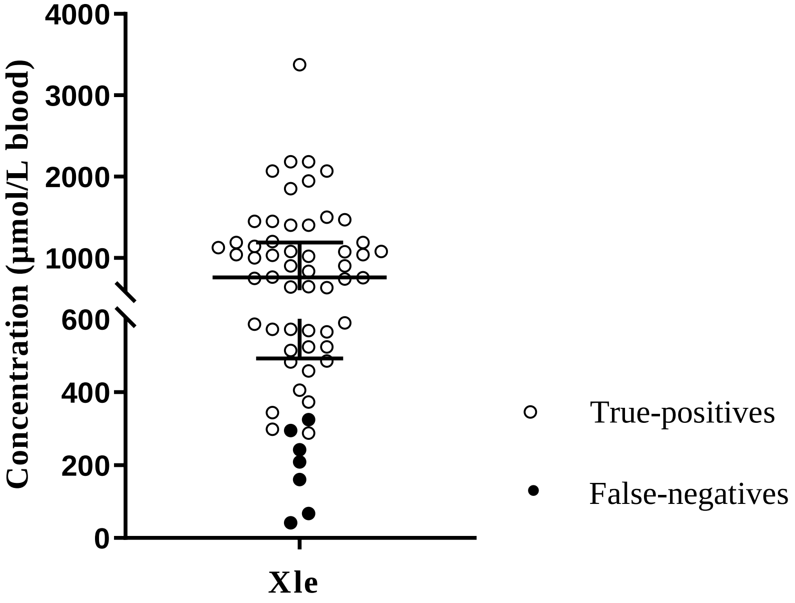

Figure S3 Total leucine (Xle) concentrations (median and IQR) measured in MSMS NBS in MSUD patients, including both true-positive and false-negative literature results
